# Genetic characterization of the cell-adapted PanAsia strain of foot-and-mouth disease virus O/Fujian/CHA/5/99 isolated from swine

**DOI:** 10.1186/1743-422X-7-208

**Published:** 2010-08-31

**Authors:** XingWen Bai, HuiFang Bao, PingHua Li, Pu Sun, WenDong Kuang, YiMei Cao, ZengJun Lu, ZaiXin Liu, XiangTao Liu

**Affiliations:** 1 National Foot-and-Mouth Disease Reference Laboratory, State Key Laboratory of Veterinary Etiological Biology, Key Laboratory of Animal Virology of the Ministry of Agriculture, Lanzhou Veterinary Research Institute, Chinese Academy of Agricultural Sciences, No. 1 Xujiaping, Yanchangbao, Lanzhou, Gansu 730046, PR China

## Abstract

**Background:**

According to Office International Des Epizooties (OIE) Bulletin, the PanAsia strain of Foot-and-Mouth Disease Virus (FMDV) was invaded into the People's Republic of China in May 1999. It was confirmed that the outbreaks occurred in Tibet, Hainan and Fujian provinces. In total, 1280 susceptible animals (68 cattle, 1212 swine) were destroyed for the epidemic control.

To investigate the distinct biological properties, we performed plaque assay, estimated the pathogenicity in suckling mice and determined the complete genomic sequence of FMDV swine-isolated O/Fujian/CHA/5/99 strain. In addition, a molecular modeling was carried out with the external capsid proteins.

**Results:**

The pathogenicity study showed that O/Fujian/CHA/5/99 had high virulence with respect to infection in 3-day-old suckling-mice (LD_50 _= 10^-8.3^), compared to O/Tibet/CHA/1/99 (LD50 = 10^-7.0^) which isolated from bovine. The plaque assay was distinguishable between O/Fujian/CHA/5/99 and O/Tibet/CHA/1/99 by their plaque phenotypes. O/Fujian/CHA/5/99 formed large plaque while O/Tibet/CHA/1/99 formed small plaque.

The 8,172 nucleotides (nt) of O/Fujian/CHA/5/99 was sequenced, and a phylogenetic tree was generated from the complete nucleotide sequences of VP1 compared with other FMDV reference strains. The identity data showed that O/Fujian/CHA/5/99 is closely related to O/AS/SKR/2002 (94.1% similarity). Based on multiple sequence alignments, comparison of sequences showed that the characteristic nucleotide/amino acid mutations were found in the whole genome of O/Fujian/CHA/5/99.

**Conclusion:**

Our finding suggested that C275T substitution in IRES of O/Fujian/CHA/5/99 may induce the stability of domain 3 for the whole element function. The structure prediction indicated that most of 14 amino acid substitutions are fixed in the capsid of O/Fujian/CHA/5/99 around B-C loop and E-F loop of VP2 (antigenic site 2), and G-H loop of VP1 (antigenic site 1), respectively. These results implicated that these substitutions close to heparin binding sites (E136G in VP2, A174 S in VP3) and at antigenic site 1 (T142A, A152T and Q153P in VP1) may influence plaque size and the pathogenicity to suckling mice.

The potential of genetic characterization would be useful for microevolution and viral pathogenesis of FMDV in the further study.

## Background

Foot-and-mouth disease (FMD) is an acute, highly contagious viral disease of cloven-hoofed animals, mostly cattle, swine, sheep and goats, leading to severe economic losses due to reduction in livestock production and restriction of trade on animals and animal products. The etiological agent, foot-and-mouth disease virus (FMDV), belongs to the genus *Aphthovirus *of the Picornaviridae family. Seven distinct serotypes of FMDV (O, A, C, Asia1 and SAT1-3), with numerous subtypes in each serotype, are not distributed equally around the world [1-3].

The genome of FMDV is composed of a positive-sense, single-stranded RNA that is approximately 8,200 nucleotides (nt) in length. The viral RNA contains 5'-untranslated region (5'-UTR), a single long open reading frame (ORF), and 3'-untranslated region (3'-UTR), followed by a poly(A) tail at its 3' end [4]. There is a small viral protein, VPg (3B), covalently linked to the 5' end of the genomic RNA [5]. The viral ORF encodes a single polyprotein, which is subsequently cleaved into multiple mature proteins (Lab/Lb; VP4, VP2, VP3, and VP1; 2A, 2B, 2C, 3A, 3B1-3, 3C, and 3D) by viral proteases (L^pro^, 2A, and 3C^pro^) [6,7]. The viral capsid comprised of 60 copies of the four structural proteins termed VP4 (internal), VP2, VP3 and VP1, surrounds the RNA. The 5'-UTR, consists of the S-fragment, poly(C) tract, 2-4 pseudoknots (PKs), a *cis*-acting replication element (*cre*), and an internal ribosome entry site (IRES). This region is predicted to display complex secondary structures, and contains several genetic elements necessary to control essential function in viral replication and gene expression [8]. The 3'-UTR, a region of about 90 nt of heterogeneous sequence, is a highly ordered structure, and stimulate the cap-independent translation and likely affect other aspects of viral infection cycle [9,10].

During 1997-2002, outbreaks of FMD caused by FMDV serotype O, occurred in the countries and districts of East Asia (EA) and the Far East [11]. The O/YUN/TAW/97 strain, a member of the Cathay topotype, containing the deletion of codons 93 to 102 in 3A coding region, is associated with the porcinophilic properties that caused a catastrophic outbreak of FMD in Taiwan [12-14]. The O/AS/SKR/2002 strain, a member of the PanAsia lineages, contains an intact 3A coding region of the virus that developed typical lesions of FMD with highly virulent and contagious in pigs but very limited in cattle [15]. In addition, pigs infected experimentally with another PanAsia strain of FMDV (O/JPN/2000) showed typically clinical signs of FMD, but the disease in Japanese black cattle was atypical, no clinical signs in an infection of Holstein cattle, and sheep and goats were not susceptible [16]. Comparison of amino acid sequence of structural proteins of two different plaque phenotypes in O/JPN/2000 strain, revealed that two substitutions existed in VP2 (133rd) and VP3 (56th) [17,18]. These substitutions may influence heparin-binding feature and in the attenuation of this virus in the natural host. Unfortunately, these two mutations close to heparin interacting regions cannot account for the characteristics of the PanAsia strains isolated from China (as detailed in Results & Discussion).

Here, we first report the cell-adapted PanAsia strain (O/Fujian/CHA/5/99) of FMDV isolated from swine in Fujian province of China in 1999, perform plaque assay and estimate the pathogenicity in suckling mice, determine the complete genomic sequence for comparison with O/YUN/TAW/97 and 14 reference strains of PanAsia lineages. Furthermore, we model the three dimensional structure of the predominant conformation in the surface FMDV capsid proteins to mimic the probable altered receptor-ligand interactions, triggered by substitutions of residues in VP1, VP2 and VP3.

## Results

### Comparison of plaque phenotypes and infectivity of O/Fujian/CHA/5/99, and O/Tibet/CHA/1/99 strain

**Figure 1:**
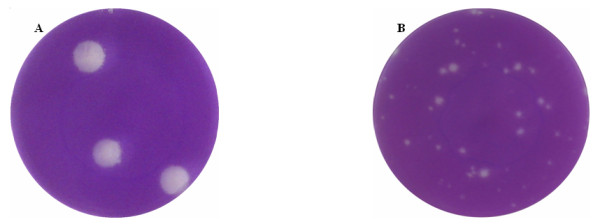
**Plaque phenotypes of FMDV O/Fujian/CHA/5/99 and O/Tibet/CHA/1/99 fixed with cold acetone/methanol and stained with 0.2% crystal violet 48 h post-incubation on BHK-21 cells**. O/Fujian/CHA/5/99 formed clear, large plaque (A), while O/Tibet/CHA/1/99 formed small plaque, shaped a fringe of snowflakes (B).

FMDV O/Fujian/CHA/5/99 strain of the 6th passage producing obvious cytopathic effect (CPE) was adapted to BHK-21 cells, and formed clear large plaque. However, the FMDV bovine-isolated O/Tibet/CHA/1/99 strain formed small plaque shaped a fringe of snowflakes (Fig. 1). The virus titres of O/Fujian/CHA/5/99 (1.5 × 10^7 ^PFU/ml) was no significant different from O/Tibet/CHA/1/99 (2.0 × 10^7 ^PFU/ml). However, the pathogenicity in suckling mice of O/Fujian/CHA/5/99 was distinguishable from that estimated with O/Tibet/CHA/1/99. The LD_50 _value was 10^-8.3 ^for O/Fujian/CHA/5/99 compared to 10^-7.0 ^for O/Tibet/CHA/1/99.

### The complete genomic sequence of O/Fujian/CHA/5/99 strain

The genome sequence of the O/Fujian/CHA/5/99 strain is 8,172 nt (excluding the poly(C) tract and the poly(A) tail) in length including a 1,081-nt 5'-UTR which is divided into S (366 nt), PKs (219 nt), *cre *(54 nt), and IRES (442 nt), a 6,999-nt ORF that encodes 2,332 amino acids terminating at a "TAA" stop codon, and a 92-nt 3'-UTR. All sequences were unique and comprised the complete genome, excluding 36 primer orderly determined nucleotides [22 nt (S+ primer) at the 5' end of the viral genome, 7 and 8 nt (Pan/S-, Pan/I+ primers) on either side of the poly(C) tract] (Table 1). The full-length genomic sequence of FMDV O/Tibet/CHA/1/99 strain has been determined and submitted to GenBank (accession NO, AF506822) by Zhang et al (2003) [19].

**Table 1:** Primers used for amplification of the complete genomic sequence of FMDV O/Fujian/CHA/5/99 strain[a]

Primers	Nucleotide sequence (5'-3')	Position
S+	TTGAAAGGGGGCGCTAGGGTCT	1-22
Pan/S-	AAAACTTAGGGGGGGGGGGGGGGGGGGGTGAAAGG	362-376[b]
Pan/I+	CCTTTCACCCCCCCCCCCCCCCCCCCCTAAGTTTT	362-376[b]
L3	GTTCTGGTACTGCTGCATGTAG	1759-1780
Pan/204	ACCTCCAACGGGTGGTACGC	1544-1563
NK61	GACATGTCCTCCTGCATCTG	3998-4017
P211	CGCTGCCTACCTCCTTCAAT	3726-2745
P222	ACTATCTCAAAGTTTTCCTTCAG	5519-5541
Pan/201	ACGAGAAGGTGTCGAGCCACC	5322-5342
Pan/205	TGTACGCGCTCCTCAACATCTC	6687-6708
D3+	CAAGGCGGGTTACTGTGGAGGAG	6502-6524
Dnn-	GCGGCCGCCATATGTTTTTTTTTTTTTTTTTTTTTTTTTTTTTTTTTTTTTTTTT	3' end

[a] The primer pairs used in this study were designated based on the FMDV O/Tibet/CHA/1/99 strain [19].

[b] The primers position was calculated without 20 G/C.

### Nucleotide sequence alignments and amino acids comparison

A detailed examination of the mutations in the whole genome of the O/Fujian/CHA/5/99 strain was based on multiple sequence alignments (Table 2). The S-fragment is 366 nucleotides in length at the 5' terminus of the viral genome, which is predicted to form a large hairpin structure. Nucleotide transitions and deletions were found at positions T82C, T84C, C105T, C119T, T138C, A139G, T145C, T147C, C155T, C160T, C182T (peak-loop), T222C, C238T, C280T, T288C, T327C, C345T, and T199, A200 in O/Fujian/CHA/5/99, which compared to reference strains of PanAsia lineage. Downstream of the poly(C) tract, there is a stretch sequence of highly tolerant to changes, containing four PKs in structure of O/Fujian/CHA/5/99 (positions -1 to +218) for the maintenance of biological function. Substitutions were observed at positions T26C, A52T and T114C; A51G, C121T (including O/AS/SKR/2002); T132C and T193C (including O/JPN/2000) in O/Fujian/CHA/5/99. Notably, a 43-nt deletion started at postion 53 downstream of the poly(C) tract in O/YUN/TAW/97 strain was determined [20], resulting in the pseudoknot 2 deletion. The conserved AAACA sequence in *cre *is required for viral RNA genome replication, while A30G, T33C distinctively located at this hairpin loop of O/Fujian/CHA/5/99 and O/AS/SKR/2002. The IRES element consists of a five structural domains, where several conserved motifs were identified [8]. In addition, the formation of a helical structure around positions 67 (G) and 275 (C) located at the base of domain 3 is needed for efficient internal initial of FMDV RNA translation [21]. Here, the substitution of C275T in O/YUN/TAW/97, O/AS/SKR/2002 and O/Fujian/CHA/5/99 strains, may induce a reorganization of the whole element with important consequences for IRES function in cattle? The other variations were present at positions T55C (domain 2), C228T (domain 3), T312C and C389T (domain 4), T423C, C436T, T437C, and A428C, 442A in O/Fujian/CHA/5/99.

**Table 2:** Amino acid differences in the whole genome of FMDV O/Fujian/CHA/5/99 as compared to O/Tibet/CHA/1/99

Untranslated region		Nucleotide mutation[a]	Secondary structure[c]	Polyprotein	Amino acid substitution[b]	Secondary structure[c]
5'-UTR	S	T82C		Lpro	A25T	
		T84C			Q26R	
		C105T			T55A	
		C119T			F68L	
		T138C			Y73S	
		A139G			P75H	
		T145C			D81S	
		T147C			K144Q	
		C155T			Q146H	
		C160T		VP2	G72S	B-C loop, antigenic site 2
		C182T			E136G	E-F loop
		T199-			K175R	G-H loop
		A200-			F214L	C terminus
		T222C		VP3	A174S	G-H loop
		C238T		VP1	Y72C	βD strand
		C280T			T96A	E-F loop
		T288C			G137D	G-H loop, close to antigenic site 1
		T327C			T142A	G-H loop, antigenic site 1
		C345T			A152T	G-H loop, antigenic site 1
	PKs	T26C			Q153P	G-H loop, antigenic site 1
		A51G			I168V	H-I loop
		A52T			A199T	C terminus, antigenic site 1
		T114C			L212S	C terminus
		C121T		2B	S5A	
		T132C			K48R	
		T193C		2C	K64E	
	*cre*	A30G			V92A	
		T33C			I241T	
	IRES	T55C	Domain 2		S312N	
		C228T	Domain 3	3A	I3V	
		C275T	Domain 3		H31C	
		T312C	Domain 4		I34V	
		C389T	Domain 4		I42V	
		T423C			E57D	
		A428C			I72M	Transmembrane domain
		C436T			M85T	
		T437C			A89V	
		-442A			N91D	
3'-UTR		C32T			I94T	
		A91G			T100A	
					E108G	
					N112S	
					K144E	
					E148G	
				3B1	K18R	
				3Cpro	R196K	
				3D	H27Y	
					K42Q	
					G62E	
					N63D	
					T98I	
					Q210R	
					R234K	
					R440W	

[a] The number gives the nucleotide position independently for each element of untranslated region (5'-UTR and 3'-UTR), according to FMDV O/Tibet/CHA/1/99 strain (accession NO, AF506822). The first letter corresponds to the nucleotide found in O/Tibet/CHA/1/99; -, nucleotide deletion.

[b] Single letter amino acid code is used. Position of amino acid residues is independently numbered for each protein from the amino terminus to the carboxyl terminus.

[c] Secondary structure assignments are as described previously [8,14,27,48,52,53].

**Figure 2:**
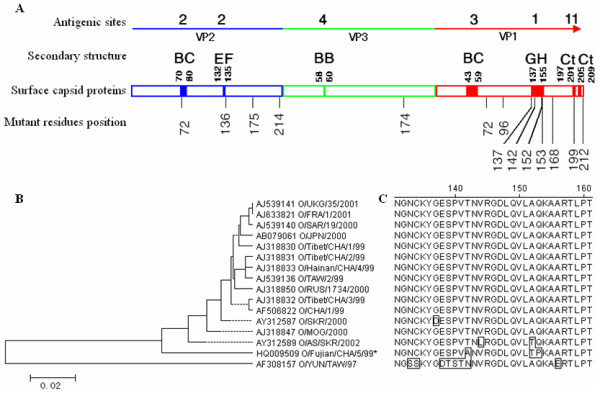
**Scheme of the location of antigenic sites in surface proteins of FMDV serotype O (A), phylogenetic tree generated from the VP1 nucleotide sequences of FMDV O/Fujian/CHA/5/99 and 15 reference strains (B) and the amino acid sequence alignments around G-H loop (positions 131 to 161) of the VP1 protein of those isolates (C)**. Mutant residues position of FMDV O/Fujian/CHA/5/99 strain is indicated (in A). The scale bar indicates the genetic distance (in B). The different amino acids are indicated in the box (in C).

The leader (L) protein, a member of the papain-like cysteine proteinase, is located at 5' end of the ORF and contains two in-frame initiation codons (84 nt in distance, Lab/Lb), that cleaves itself from the viral polyprotein [22] acting as a *trans*-proteinase and initiation factor eIF4G at G_479_/R_480 _resulting the shut-off of host protein synthesis [23]. 51 D, 148 H and 164 D were the active site residues, by playing a essential role in substrate binding [24,25]. It's also an important determinant of virulence in animals [26]. The amino acid sequence identities of O/Fujian/CHA/5/99 with reference PanAsia strains and O/YUN/TAW/97 was 92.0%-94.5% and 88.1%, respectively. The variable substitutions appeared in three distinct regions (A25T, Q26R at N-terminus; T55A, F68L, Y73 S, P75 H and D81 S on the C-terminal side of 51C; K144Q and Q146 H on the N-terminal side of 148H). VP4 is the most conserved FMDV protein. There was 100% homology in amino acid sequence between O/Fujian/CHA/5/99 and reference PanAsia strains. The amino acid sequence alignments of VP2 and VP3 showed that the specific substitutions of O/Fujian/CHA/5/99 existed at the residues E136G, K175R and F214L in VP2, and A174 S in VP3, respectively (Table 2). The 136th in VP2 and 174th of VP3 are very close to their respective heparin interacting regions (residues 134th, 135th in VP2, and 173rd in VP3, respectively). A phylogenetic tree was generated from VP1 nucleotide sequence alignment of 16 FMDV which caused outbreaks of FMD in EA and the Far East in 1997-2002 (Fig. 2). The identity data of VP1 showed that the O/Fujian/CHA/5/99 strain is clustered in the PanAsia lineage and closely related to O/AS/SKR/2002 (94.1% similarity). Furthermore, comparison of the amino acid sequences in VP1 of O/Fujian/CHA/5/99 and O/Tibet/CHA/1/99 indicated that 9 substitutions were found at residues Y72C, T96A, G137 D, T142A, A152T, Q153P, I168V, A199T and L212 S of O/Tibet/CHA/5/99 (Table 2). Most of these substitutions were present in C-terminal segment of VP1, in particular in G-H loop (antigenic site 1). The important integrin-binding RGD motif (145-147 residues), RGD+1, RGD+2 and RGD+4 were conserved for virus reception and pathogenesis in these FMDV strains (Fig. 2).

**Figure 3:**
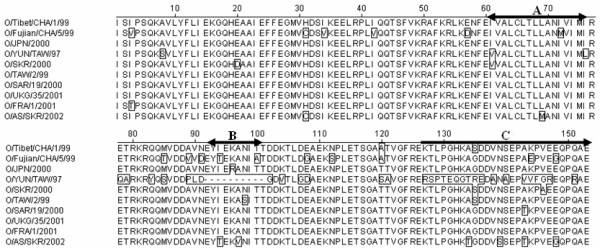
**Multiple sequence alignment of amino acid sequences of the 3A coding region of 10 FMDV strains**. The transmembrane domain contained amino acid substitutions at positions I61V and I76L (O/YUN/TAW/97), L69 M (O/AS/SKR/2002) and I72 M (O/SKR/2000 and O/Fujian/CHA/5/99) (A). The 93-102 amino acid deletions harbored in 3A of the porcinophilic phenotype of O/YUN/TAW/97 (B). The highly variable C-terminus was predicted probably due to the conformation of three-dimensional structure for 3A function (C).

In non-structural protein regions, we also found the highest degree of sequence conservation in 2A, 2B, 2C, 3B, 3C and 3 D that it was predicted probably due to their functions or interaction with host factors. The characteristic amino acid mutations occurred at residues S5A, K48R in 2B; K64E, V92A, I241T, S312N in 2C; K18R in 3B1; R196K in 3C; and H27Y, K42Q, G62E, N63 D, T98I, Q210R, R234K and R440W in 3 D of O/Fujian/CHA/5/99, respectively (Table 2). Comparison of 3A protein sequences showed that O/Fujian/CHA/5/99 contains a full-length 3A coding region, whereas the 93-102 amino acid deletions harbored in 3A of O/YUN/TAW/97 (Fig. 3). I72 M was present in transmembrane domain (positions 60-76) as previously described [14]. The other 14 amino acid substitutions were identified at positions I3V; H31C and I34V; I42V and E57D; M85T, A89V and N91D; I94T and T100A; E108G, N112 S, K144E and E148G in O/Fujian/CHA/5/99 (Table 2), which predicted to undergo positive selection of viral evolution. These data suggested that the variability of 3A may be highly informative for molecular epidemiological studies.

The 3'-UTR of O/Fujian/CHA/5/99, a region of 92 nt with high tolerant changes (72.8%-95.7% similarity) following the ORF termination codon, contains a "Y" shape of RNA which is required for its function, where the nucleotide changes of C32T and A91G were observed (Table 2).

### Molecular modeling

**Figure 4:**
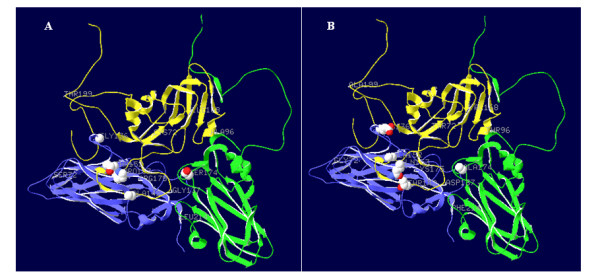
**Locations of 14 amino acid differences (L212 S not shown) mapped in the surface capsid proteins of FMDV O/Fujian/CHA/5/99 (A) and O/Tibet/CHA/1/99 (B)**. The potential critical amino acid residues were measured at positions 136 in VP2; 174 in VP3; 142, 152, 153 in VP1, which are represented as globe in VP2 (blue), VP3 (green) and VP1 (yellow), respectively.

We have identified that O/Fujian/CHA/5/99 and O/Tibet/CHA/1/99 were differed in the amino acid sequence of VP2, VP3 and VP1 (Table 2). By using the atomic coordinates obtained by X-ray crystallography of FMDV O_1_BFS, six mutations which are clustered the position occupied by the G-H loop of VP1 fixed in the capsid of O/Fujian/CHA/5/99 were determined (K175R in VP2, A174 S in VP3, G137 D, T142A, A152T and Q153P in VP1). E136G substitution in VP2 was measured close to antigenic site 2 within E-F loop; Y72C, T96A and A199T substitutions in VP1 are located at βD-strand, E-F loop and antigenic site 1 of C-terminus, respectively. In addition, G72 within B-C loop (antigenic site 2) of VP2 is fixed in O/Tibet/CHA/1/99, and I168 of VP1 mapped in H-I loop (Fig. 4). As stated previously [18,27,28], these substitutions around heparin binding sites and antigenic site 1 on the viral capsid may influence plaque size and the pathogenicity to suckling mice.

## Discussion

The PanAsia strain of FMDV serotype O originated in India no later than 1982 [29]. It has been the most dominant outbreak strain in the recent years and distributed around the world in over 24 countries [30]. Towards the west, the virus spread into Saudi Aribia (1994), then emerged as the pandemic virus circulating in Middle East, Middle East-South Asia region and into European countries such as Turkey, Greece and Bulgaria (1996) [3,11,31]. Furthermore, the virus strain even invaded into South Africa (2000) [32]. A catastrophic outbreak caused by the same viral lineage occurred in the United Kingdom in 2001, and subsequently spread into Ireland, France and the Netherlands within 1 mouth [3,32]. Towards the east, the virus spread into Nepal (1993), Bangladesh (1996), Bhutan (1998), China (1999), Japan (2000), Korea (2000) and finally invaded countries of the Far East such as Mongolia (2000) and Russia (2000) [3,11,33].

The extent to the genetic diversity of these PanAsia virus isolates accumulating over the course of FMD outbreaks with infection of susceptible animals, is contributed to the understanding of the occurrence of phenotypic changes in cultured cells and alteration in host tropism. Here, a gradual accumulation of nucleotide/amino acid mutations were observed in O/Fujian/CHA/5/99 evolving in FMDV populations. The radical ambiguities of convergent evolution will potentially affect the functional and/or structural features involved in 5'-UTR and 3'-UTR of FMDV, respectively. The S-fragment located at the 5' terminus of the FMDV genome may play a role in the switch from translation to replication [34]. The variable nature of PKs was documented that it can be used along with the 3A-based phylogenetic tree for genetic analysis of FMDVs (data not shown). Mutations in the AAACA motif and the stem region of the *cre *element significantly reduced replication of FMDV genome [35], suggesting that two substitutions (positions 30th, 33rd) located at the loop within this structure of O/Fujian/CHA/5/99 may induce decreasing for RNA replication *in vivo*. Deletion, insertion and substitutions (the majority of which were transitions) probably lead to changes in the organization of the IRES structure, resulting in modulated its activity for internal initiation of translation [8]. The structure of 3'-UTR could affect the infectivity of FMDV due to RNA-RNA and RNA-protein interactions [8].

In the present study, George *et al. *(2001) [36] has discussed that few unusual variations in the L protein may reflect its role in either RNA-RNA or RNA-protein interactions that specifically enhanced IRES-dependent translation. By sequencing the structural proteins of O/Fujian/CHA/5/99, we have provided the first homology analysis of the plaque-purified PanAsia strain of FMDV isolated from swine in China. In spite of the evidence generated from O/JPN/2000 [18] and studies determined by Sa-carvalho *et al. *(1997) [37], our analysis of O/Fujian/CHA/5/99 and 9 reference strains of FMDV indicated that all of these viruses display 133 D in VP2 (excluding D133 S in VP2 of O/YUN/TAW/97) and 56 H in VP3. Chinese Yellow cattle and native cattle infected experimentally with the FMDV O/Taiwan/99 strain showed no clinical signs. However, pigs infected with O/Taiwan/99 developed typical disease [33]. In Korea, the isolated virus (O/SKR/2000) infected Holstein cattle caused typical vesicles in the field, but did not develop typical vesicular lesions on the foot in animal experiments (OIE, 2000). The 174th amino acid in VP3 substitution was presumably provided as a practical explanation for attenuated virulence of these viruses in cattle (Table 2). Meanwhile, Y79 H (O/JPN/2000 and O/YUN/TAW/97), E136G (O/AS/SKR/2002 and O/Fujian/CHA/5/99) and F214L (O/YUN/TAW/97, O/AS/SKR/2002 and O/Fujian/CHA/5/99) in VP2, T56I (O/YUN/TAW/97 and O/AS/SKR/2002), N85 D (O/YUN/TAW/97), T96A (O/AS/SKR/2002 and O/Fujian/CHA/5/99), T142N/A (O/YUN/TAW/97 and O/Fujian/CHA/5/99, respectively) and A152T (O/AS/SKR/2002 and O/Fujian/CHA/5/99) in VP1 may be associated with bovine attenuation of these viruses. Y79 H within βC strand and E136G within E-F loop of VP2, T142N/A and A152T within G-H loop of VP1 are exposed on the surface of the viral capsid. The direct induction of capsid alterations in the cell attachment sites may influence virus interaction with cellular receptor for FMDV adaptation to cells in culture and mild pathogenicity [38,39].

The degree of conservation was somewhat higher for 2A, 2B, 2C, 3B1-3 and 3C, and the impact of adaptive positive selection at the amino acid level on these non-structural proteins has been found by identified genome regions of 10 FMDV isolates involved in genetic diversity. To date, these included N1 S (O/JPN/2000), D3N(O/TAW/2/99) and S13P (O/AS/SKR/2002) in 2A; I18V (O/YUN/TAW/97 and O/AS/SKR/2002) in 2B; Q164 H (O/AS/SKR/2002) and I241T (O/Fujian/CHA/5/99) in 2C (nearly the conserved motifs D_160_DLG_163 _and N_243_KLD_246_, respectively); K18E (O/JPN/2000) in 3B1 and V17A (O/YUN/TAW/97 and O/AS/SKR/2002) in 3B2. 3A contains residues predicted to undergo positive selection with respect to infection in guinea pigs [40]. Deletions in 3A have been associated with altered host range in the hepatoviruses [41], rhinoviruses [42], enteroviruses [43], and aphthoviruses [12-14,44]. This deletion cannot be found in the 3A region of O/Fujian/CHA/5/99, which has high similarity with the other PanAsia strains (91.7%-92.6% in nucleotide sequences and 86.9%-89.5% in amino acid sequences, respectively). I61V and I76L (O/YUN/TAW/97), L69 M (O/AS/SKR/2002) and I72 M (O/Fujian/CHA/5/99) in transmembrane domain were observed (Fig. 3). The highly variable C-terminal half (positions 117 to 143) in the 3A coding region of O/YUN/TAW/97, form a short α-helix (Zhang *et al*., unpublished data, 2007). A previously described FMDV mutant 3D^pol ^with amino acid replacement D338A in the NTP-binding domain (the peptide motif Y_336_GDD_339_) destroyed the viral polymerase activity [45] suggesting that although 3D^pol ^is more tolerate of substitutions at most positions, conservation of the tertiary structure is likely to be necessary for its function. These observations implied that dramatic alteration in these regions contributed to properties of these proteins and the fitness of dynamic mutant distributions, though the pathogenicity of O/Fujian/CHA/5/99 in cattle is not clear.

Thus, further investigations should aim to O/Fujian/CHA/5/99 infected normal hosts in animal experiments and the finding of molecular basis for the derivation of genetic mutants by utilizing reverse genetics. These studies may help us to clarify how is it that the mutations responsible for genetic diversity and antigenic drift have a moderate effect on the interactions of FMDV to its cellular receptors and in responses to selective constraints.

## Conclusion

Our studies found very different phenotypes and pathogenicities between FMDV O/Fujian/CHA/5/99 strain and O/Tibet/CHA/1/99. The distinct biological properties are the results of error-prone replication of genome during viral life cycles. Our findings indicate that nucleotide and amino acid mutations were present in the whole genome of O/Fujian/CHA/5/99, as compared to O/Tibet/CHA/1/99. The great majority of these mutations associated with the effect of viral fitness in physical and biological environment. Advantageous mutations fixed on the viral genome of O/Fujian/CHA/5/99 may be essential contributed to FMDV adaptation of susceptible animals in the field. Consequently, future study can be interested in these predictions for the understanding of viral populations, genetic variability and its biological implications.

## Methods

### Cells, sample collection and virus isolation

Baby hamster kidney (BHK-21) cells were maintained at 37°C in Dulbecco's modified Eagle's medium (DMEM, Gibco) containing 10% fetal bovine serum (FBS, Hyclone). The sample of vesicles on hoof was collected from swine, which showed clinical symptoms of FMD, in Fujian province of China (OIE, May 1999). The grinding suspension (1/10, w/v) was prepared in phosphate buffered saline (PBS) containing the antibiotics penicillin (100 U/ml) and streptomycin (0.1 mg/ml), overnight at 4°C, clarified by centrifugation at 2,000 × g for 10 min, sterilized by using 0.45 μm filter unit (Millipore), and the virus was propagated on BHK-21 cells as described previously [46]. The isolated virus adapted to BHK-21 cells was designated O/Fujian/CHA/5/99 strain. The FMDV O/Tibet/CHA/1/99 strain isolated from bovine in Tibet of China was used in this work and conserved in national foot-and-mouth disease reference laboratory.

### Plaque assay and the pathogenicity in suckling-mice

Confluent BHK-21 cell cultures in 6-well plates were prepared for plaque-forming assay. The serial 10-fold dilutions of viruses were inoculated 200 μl per well. After 1 h of incubation at 37°C in 5% CO2, 2 ml overlay medium containing 0.6% Gum and 1% FBS was added and cultured for 48 h under the same conditions. Subsequently, the BHK-21 cells were washed three times with PBS (pH 7.5), then fixed with cold acetone/methanol for 20 min at -20°C, and stained with 0.2% crystal violet for 30 min at room temperature. Finally, we were able to observe plaque morphology and calculate virus titres by plaque-forming units (PFU) from the infected BHK-21 cell cultures.

Serial ten-fold diluted viruses were prepared in DMEM containing 2% FBS, and the pathogenicities were titrated by intraperitoneal inoculation of 3-day-old suckling-mice in groups of five animals each with 0.2 ml of virus dilutions. The suckling mice were observed for 72 h after infection and the 50% lethal dose (LD_50_) was determined by the method of Reed and Muench (1938) [47].

### Sequencing and genetic characterization

Rneasy Mini Kit (Qiagen) RNA extraction was performed as the manufacture's protocol. 4 μl 5 × AMV buffer, 4 μl 10 mM dNTP, 10 μl RNA, 1 μl 50 pmol/L anti-sense genome specific primers (Pan/S-, L3, NK61, P222, Pan/205, and Dnn-, respectively; Table 1), 1 μl AMV (TaKaRa) mix was incubated at 42°C for 1 h and then on ice for 3 min. After completion of the reverse transcript (RT) reaction, six overlapping PCR fragments covering the viral genome were amplified from each sample by using specific primer sets (set 1, S+ and Pan/S-; set 2, Pan/I+ and L3; set 3, Pan/204 and NK61; set 4, P211 and P222; set 5, Pan/201 and Pan/205; set 6, D3+ and Dnn-; Table 1) with LA Taq DNA polymerase (TaKaRa). The target PCR products were cleaned up using Wizard^® ^Gel and PCR Clean-Up System (Promega) and cloned into pGEM^®^-T Vector (Promega). Cycle sequencing reaction were performed with fluorescent BigDye chain terminators (Applied Biosystems), followed by resolution on an ABI Prism 310 genetic analyzer (Applied Biosystems).

The complete genetic sequences were assembled using SeqMan (DNAStar). Multiple sequence alignment was analyzed using MegAlign (DNAStar) to construct a phylogenetic tree. MegAlign was also used for the genomic analysis of nucleotide mutations in 5'-UTR and 3'-UTR, and amino acid substitutions in leader (L) protein, structural proteins and non-structural proteins. The atomic coordinates of FMDV crystallized for O_1_BFS [48-51] were used to model the conformations, and the structures of FMDV O/Tibet/CHA/1/99 and O/Fujian/CHA/5/99 strains were optimized by placing substituted amino acids which exist in the external surface of capsid, in their standard conformations.

## Competing interests

The authors declare that they have no competing interests.

## Authors' contributions

XWB participated in planning of the study and carried out the phylogenetic analysis and drafted the manuscript. HFB performed plaque assay. PHL and PS were involved in the determination of nucleotide sequences. WDK carried out molecular modeling. YMC and ZJL participated in the experiments of the pathogenicity in suckling mice. XTL and ZXL collected the field isolates and delivered background information, and ZXL conceived the study. All authors reviewed and approved the final manuscript.
